# Comparing thermographic heat signatures with joint inflammation detected by ultrasound at the knees in patients with rheumatoid arthritis

**DOI:** 10.1038/s41598-025-17901-6

**Published:** 2025-08-30

**Authors:** York Kiat Tan, Gek Hsiang Lim

**Affiliations:** 1 Department of Rheumatology and Immunology, Singapore General Hospital, Outram Road, Singapore, 169608 Singapore; 2 Duke-NUS Medical School, Singapore, Singapore; 3 Health Services Research Unit, Singapore General Hospital, Singapore, Singapore

**Keywords:** Rheumatoid arthritis, Ultrasound, Thermography, Joints, Synovitis, Knee, Diseases, Rheumatic diseases

## Abstract

Joint inflammation at the bilateral knees of patients with rheumatoid arthritis (RA) was assessed using thermal imaging (TI) in comparison with ultrasonography. The minimum (T-min), maximum (T-max) and average (T-avg) temperatures at the lateral, anterior and medial knee aspects were correlated with ultrasound-detected grey-scale (GS) and power Doppler (PD) joint inflammation. The use of TI in identifying ultrasound GS score ≥ 2 and PD score > 0 were assessed using receiving operating characteristic (ROC) curve analysis. A subset of baseline thermograms were re-read > 2 weeks apart for intra-observer reliability testing using intra-class correlation coefficient (ICC) analysis. In this cross-sectional study (*n* = 95 RA patients), 570 thermograms were obtained while 190 knees were scanned by ultrasound. All TI parameters (T-min, T-max and T-avg) correlated significantly (*P* < 0.05) with ultrasound PD and GS scores (correlation coefficient ranged from 0.21 to 0.49). Using TI parameters to identify ultrasound PD score > 0 and GS score ≥ 2, the area under the ROC curves (AUCs) ranged from 0.63 to 0.82. The ICC values for the TI parameters were high (ranging from 0.997 to 0.999). Thermographic heat signatures are reflective of ultrasound-detected joint inflammation and can help discriminate ultrasound PD positivity/negativity and GS joint inflammation severity at the knees in patients with RA.

## Introduction

Heat (or calor) is one of the clinical cardinal signs of inflammation, the remaining being rubor, dolor, tumor and functio laesa^1^. Infrared thermography, or thermal imaging (TI), is a contactless imaging modality that can detect infrared radiation emitted from surface(s) or object(s) and quantifying it as temperature measurements^2,3^. Rheumatological conditions such as rheumatology arthritis (RA) that activates the inflammatory cascade can produce heat signatures at the skin surfaces of the joints which can be detectable by TI^4,5^. Over the past decade, there have been numerous studies documenting the use of TI in various arthritides, including RA^1,4,6^. In RA, the use of TI has been more frequently studied at the hand/wrist of patients when compared to other joint sites^4,7–9^. A recent small-scale study among patients with RA has demonstrated that hand/wrist joints in RA patients have significantly higher temperature readings in the presence of ultrasound-detected power Doppler (PD) as well as grey-scale (GS) joint inflammation^7^. In another study (involving 31 RA patients and 51 healthy control), the temperatures at the palm and finger were found to be significantly greater among RA patients (without active inflammation) when compared to healthy controls^8^ while a separate hand study (with 49 RA patients and 30 healthy controls) found that joint temperature of RA patients was higher than healthy controls^9^. There have been fewer studies on the use of TI at larger joint sites, such as the knees of patients with RA^4,10,11^, with two previous knee studies^10,11^ demonstrating positive correlation between thermographic temperatures and ultrasound PD joint inflammation (although GS joint inflammation assessment was not included in both these studies). Ultrasound synovitis has been defined as having either PD joint inflammation score > 0 (i.e. positivity) or GS joint inflammation score ≥ 2 (i.e. at least moderate severity) in several RA studies^12–14^. Unlike previous knee studies which compared TI to PD joint inflammation only^10,11^, our present RA knee study compares the use of TI with both ultrasound PD and GS joint inflammation. Studying TI in relation to both ultrasound PD and GS joint inflammation would be necessary as both are recognised components of ultrasound synovitis in RA^12–14^. Ultrasonography and magnetic resonance imaging (MRI) are presently well-established imaging modalities for joint inflammation assessment in patients with arthritides, including RA^15,16^. However, the use of ultrasound and MRI are not without limitations^17,18^. MRI is generally too costly for wide-spread routine clinical use for joint inflammation assessment in RA while ultrasonography can be labour intensive when scanning multiple different joint sites and sonographers typically require a substantial period of training before gaining competency in musculoskeletal ultrasonography^17,18^. There is therefore a need to look for other low-cost imaging modalities that can efficiently assess joint inflammation in patients with RA amidst the busy rheumatology clinic settings. TI is well-suited for this role as it is relatively inexpensive, safe (being non-invasive and without ionizing radiation effects) and straight-forward to use; with modern thermal cameras being highly portable, compact and allowing for quick image acquisition^4,6^. At present, the use of TI as a modality for joint inflammation assessment in the RA imaging literature is much less described when compared to other more established modalities like ultrasound and MRI^4,16^. Unlike both ultrasound and MRI, thermography is not able to directly visualize and assess specific pathologies such as degree of synovial thickening and whether there are any bone erosions^4,18^. Rather, it relies on detecting the heat emitted from joint surfaces that accompanies joint inflammation in patients with RA^4^. By studying TI alongside ultrasonography, we aim to compare the former with the latter for joint inflammation assessment and to evaluate the ability of TI in identifying ultrasound-detected joint inflammation at the knees of patient with RA.

## Materials and methods

In this single-site cross-sectional study, RA patients were consecutively recruited from the outpatient rheumatology clinic of a local tertiary hospital. The RA patients included in the study were as follows: male or female patients who were aged from 21 to 99 years old and who fulfilled the 2010 EULAR/ACR RA classification criteria)^7^. Pregnant patient(s) were excluded from this study. The study received approval by the SingHealth Centralised Institutional Review Board (CIRB) (2020/2669) and conforms to the relevant research ethical guidelines. All patients provided their informed consent prior to their participation in the study.

### Patient characteristics and baseline demographic data

Patients’ 28-joint disease activity score (DAS28) performed by rheumatology nurses were recorded at baseline while other baseline patient characteristics such as the age, gender, ethnicity, disease duration and medications use (disease modifying anti-rheumatic drugs (DMARDs) and corticosteroid) were obtained from the patient’s medical records.

### Imaging assessment of the knees

**Fig. 1:**
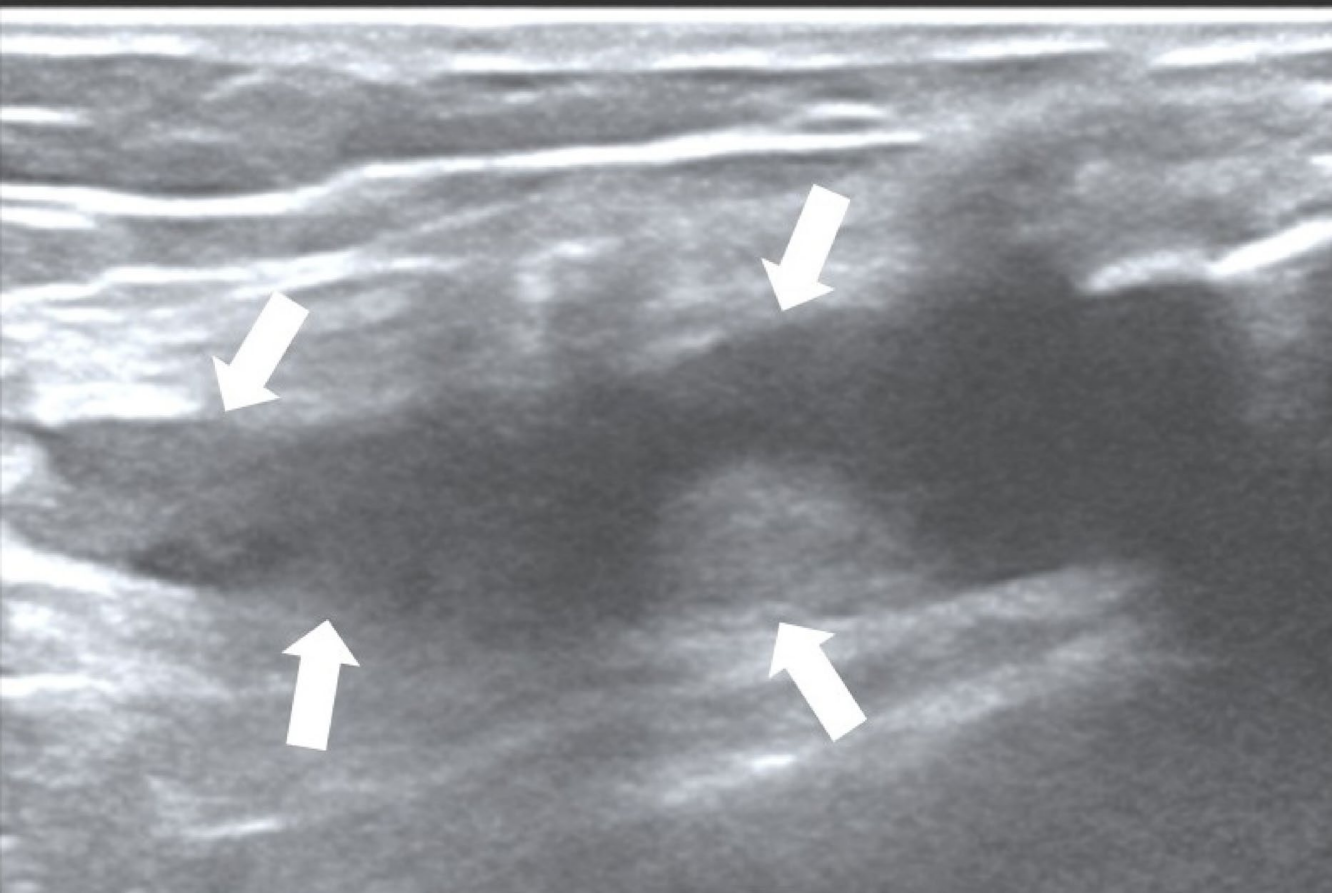
Grey-scale (GS) sonogram. Example of ultrasound imaging showing grade 2 (moderate) GS joint inflammation at the supra-patellar recess of the knee (white arrows pointing towards synovial hypertrophy) in a patient with rheumatoid arthritis.

**Fig. 2:**
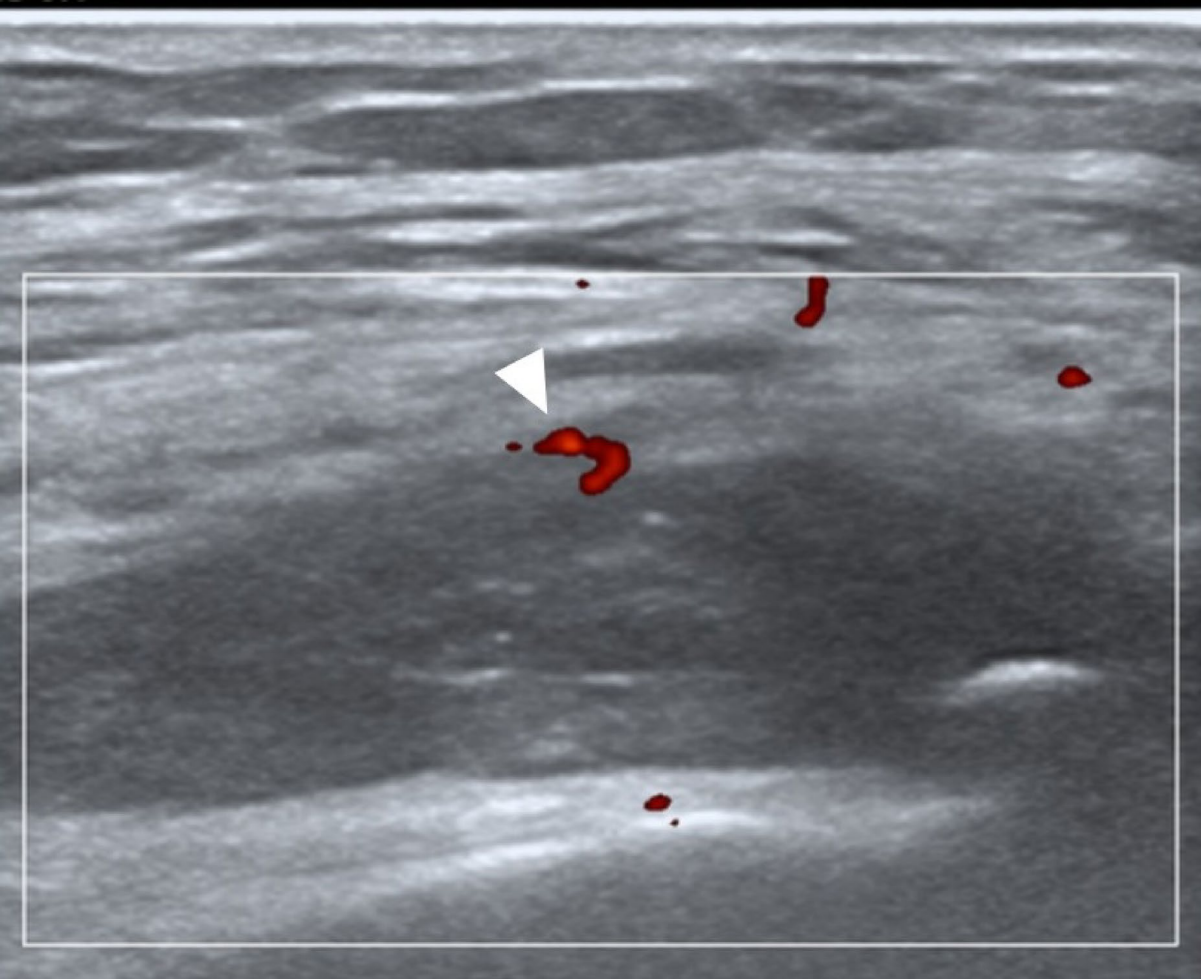
Power Doppler (PD) sonogram. Example of ultrasound imaging showing grade 1 (mild) PD joint inflammation at the supra-patellar recess of the knee (white arrowhead pointing towards PD vascularity) in a patient with rheumatoid arthritis.

Both ultrasound and TI were conducted at the same study visit for each patient. Ultrasonography at the bilateral knees’ supra-patellar recesses was performed by a rheumatologist experienced in musculoskeletal ultrasound while blinded to the findings from TI whereas thermography was carried out by a separate trained study team member while being blinded to the findings from ultrasound imaging. The Mindray M9 ultrasound machine with a L14-6Ns linear probe was utilized in this study with machine settings of pulse repetition frequency at 700 Hz and Doppler frequency at 5.7 MHz. Standardized ultrasound imaging followed the EULAR guidelines^19^. Ultrasound GS synovial hypertrophy and PD vascularity were graded semi-quantitatively (0–3) based on previously validated methods^20,21^ (see Figs. 1 and 2 which show an example of scoring ultrasound GS and PD joint inflammation, respectively.)

**Fig. 3:**
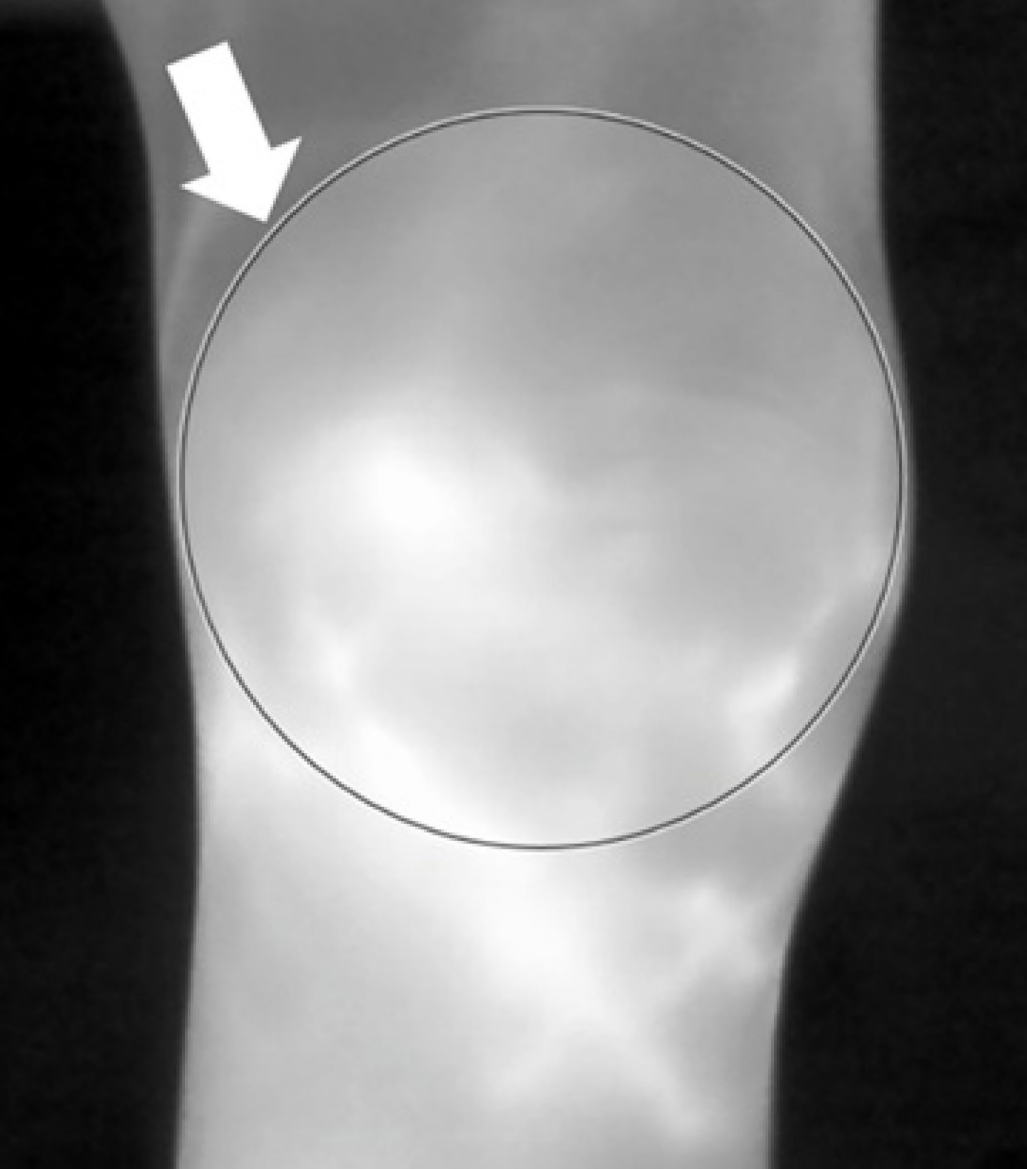
Thermal imaging showing a knee thermogram. Example of thermographic assessment (with white arrow showing the region of interest (ROI) manual segmentation) at the anterior aspect of the knee in a patient with rheumatoid arthritis. The readouts of the results of the minimum (T-min), maximum (T-max) and average (T-avg) temperatures were then obtained based on the manually segmented ROI of the thermogram.

Following methods described in the literature^4,7,22^, standardized thermography using a FLIR T640 high performance thermal camera was carried out in a draft-free room (without windows) at an ambient temperature of around 23 °C. The settings of the thermal camera are as follows: predefined emissivity value of 0.98 for skin^4^, thermal sensitivity of < 30 milli-Kelvin at 30 °C and pixel resolution of 640 × 480. The patients underwent acclimatization by resting 15 min prior to the start of TI^4,22^. For image acquisition, each knee thermogram was acquired by placing the thermal camera 50 cm away from the lateral, anterior and medial aspects of the knee. By applying the commonly utilized region of interest (ROI) manual segmentation approach^4,7^ (see Fig. 3 which shows an example of ROI manual segmentation for TI at the anterior aspect of the knee), the following thermographic temperatures readings were obtained from the ROI of each thermogram at the three aspects (lateral, anterior and medial) of the knee: the minimum (T-min), maximum (T-max) and average (T-avg) temperatures.

### Statistical analysis

At the knees bilaterally, the TI parameters (T-min, T-max and T-avg) at the three knee aspects (lateral, anterior and medial) were correlated with ultrasound outcomes (i.e. GS scores and PD scores) at the supra-patellar recesses using the Spearman’s correlation coefficient. The use of TI in identifying ultrasound PD score > 0 and GS score ≥ 2 were assessed using receiving operating characteristic (ROC) curve analysis. The optimal cut-off for the ROC analysis was chosen based on the Youden’s index (with the corresponding sensitivity (SN), specificity (SP), positive predictive value (PPV) and negative predictive value (NPV) calculated). A total of 30 baseline thermograms (from the lateral, anterior and medial aspects of 10 knees that were randomly selected) were re-read (more than 2 weeks apart) for 30 each of T-min, T-max and T-avg to test for intra-observer reliability (for single observer) using intra-class correlation coefficient (ICC) analysis. The interpretation of ICC results were as follows^22^: poor, less than 0.50; moderate, between 0.50 and 0.75; good, between 0.75 and 0.90; excellent, more than 0.90. Statistical analyses were performed using Stata 17 (StataCorp. 2021. Stata Statistical Software: Release 17. College Station, TX: StataCorp LLC).

## Results

A total of 570 knee (3 aspects per knee) thermograms were obtained while 190 knees (bilaterally) were scanned by ultrasound among 95 RA patients. The patient baseline characteristics and demographic data were as follows: mean (SD) age was 56.7 (12.9) years; 71 female patients (74.7%); 69 Chinese patients (72.6%); mean (SD) disease duration was 6.6 (5.9) months; mean (SD) DAS28 was 3.80 (1.33); all patients were on conventional disease-modifying anti-rheumatic drug (DMARDs) therapy (i.e. one or more of the following medications: hydroxychloroquine; sulfasalazine; methotrexate; leflunomide) while 66 patients (69.5%) were on oral prednisolone.

### Correlation of ultrasound and thermography at the knees

Table 1 summarizes the correlation analysis results between TI parameters and ultrasound outcomes at the bilateral knees. All TI parameters (T-min, T-max and T-avg) correlated significantly with ultrasound GS scores (correlation coefficient ranged from 0.27 to 0.49, P<0.01). Similarly, all TI parameters correlated significantly with ultrasound PD scores (correlation coefficient ranging from 0.21 to 0.43, P<0.05). Specifically, for ultrasound GS scores, the correlation coefficients with the TI parameters were as follows: 0.46, 0.38 and 0.35 for T-max at the right knee lateral, anterior and medial aspects, respectively; 0.35, 0.41 and 0.29 for T-avg at the right knee lateral, anterior and medial aspects, respectively; 0.30, 0.33 and 0.27 for T-min at the at the right knee lateral, anterior and medial aspects, respectively; 0.40, 0.43 and 0.36 for T-max at the left knee lateral, anterior and medial aspects, respectively; 0.47, 0.49 and 0.43 for T-avg at the left knee lateral, anterior and medial aspects, respectively; 0.49, 0.39 and 0.37 for T-min at the at the left knee lateral, anterior and medial aspects, respectively. Specifically, for ultrasound PD scores, the correlation coefficients with the TI parameters were as follows: 0.42, 0.33 and 0.37 for T-max at the right knee lateral, anterior and medial aspects, respectively; 0.30, 0.37 and 0.25 for T-avg at the right knee lateral, anterior and medial aspects, respectively; 0.26, 0.34 and 0.21 for T-min at the at the right knee lateral, anterior and medial aspects, respectively; 0.34, 0.39 and 0.35 for T-max at the left knee lateral, anterior and medial aspects, respectively; 0.40, 0.43 and 0.37 for T-avg at the left knee lateral, anterior and medial aspects, respectively; 0.43, 0.32 and 0.32 for T-min at the at the left knee lateral, anterior and medial aspects, respectively. Overall, some TI parameters showed a weaker correlation while others had a stronger correlation (with correlation coefficients between 0.40 to 0.50 indicating a moderate correlation) with ultrasound-detected joint inflammation. 

**Table 1:** Correlation between thermal imaging and ultrasound outcomes at the bilateral knees.

TI parameter	Knee aspect				
		PD score		GS score	
		Spearman’s rho	*P*-value	Spearman’s rho	*P*-value
Right knee					
T-min	Lateral	0.26	0.01*	0.30	0.003**
T-max	Lateral	0.42	< 0.001***	0.46	< 0.001***
T-avg	Lateral	0.30	0.004**	0.35	< 0.001***
T-min	Anterior	0.34	< 0.001***	0.33	0.001**
T-max	Anterior	0.33	< 0.001***	0.38	< 0.001***
T-avg	Anterior	0.37	< 0.001***	0.41	< 0.001***
T-min	Medial	0.21	0.04*	0.27	0.008**
T-max	Medial	0.37	< 0.001***	0.35	< 0.001***
T-avg	Medial	0.25	0.02*	0.29	0.004**
Left knee					
T-min	Lateral	0.43	< 0.001***	0.49	< 0.001***
T-max	Lateral	0.34	< 0.001***	0.40	< 0.001***
T-avg	Lateral	0.40	< 0.001***	0.47	< 0.001***
T-min	Anterior	0.32	0.002**	0.39	< 0.001***
T-max	Anterior	0.39	< 0.001***	0.43	< 0.001***
T-avg	Anterior	0.43	< 0.001***	0.49	< 0.001***
T-min	Medial	0.32	0.002**	0.37	< 0.001***
T-max	Medial	0.35	< 0.001***	0.36	< 0.001***
T-avg	Medial	0.37	< 0.001***	0.43	< 0.001***

*TI* Thermal imaging, *T-min* Minimum temperature,* T-max* Maximum temperature, *T-avg* Average temperature, *PD* Power Doppler, *GS *Grey-scale. Statistical significance:

*P<0.05, **P<0.01, ***P<0.001.

### ROC analysis at the knees

Table 2 and table 3 summarize the results of the ROC analysis for TI parameters (T-min, T-max and T-avg) in identifying PD score > 0 and GS score ≥ 2 at the knees, respectively. For the use of T-min, T-max and T-avg in identifying ultrasound PD score > 0, the area under the ROC curves (AUCs) ranged from 0.63 to 0.82 (see table 2 for the corresponding optimal cut-off, youden’s index, SN, SP, PPV and NPV results). For the use of T-min, T-max and T-avg in identifying ultrasound GS score ≥ 2, the AUCs ranged from 0.65 to 0.82 (see table 3 for the corresponding optimal cut-off, youden’s index, SN, SP, PPV and NPV results). For ultrasound PD joint inflammation (Table 2), TI parameters with AUC > 0.70 to 0.80 are as follows: right knee anterior aspect (T-avg and T-min), lateral aspect (T-max) and medial aspect (T-max); left knee anterior aspect (T-max and T-min), lateral aspect (T-max, T-avg and T-min) and medial aspect (T-max and T-min). For ultrasound PD joint inflammation (Table 2), TI parameters with AUC > 0.80 are as follows: Left anterior aspect (T-avg) and medial aspect (T-avg). For ultrasound GS joint inflammation (Table 3), TI parameters with AUC > 0.70 to 0.80 are as follows: right knee anterior aspect (T-max, T-avg and T-min) and lateral aspect (T-max and T-avg); left knee anterior aspect (T-max and T-min), lateral aspect (T-max and T-min) and medial aspect (T-max and T-min). For ultrasound GS joint inflammation (Table 3), TI parameters with AUC > 0.80 are as follows: Left anterior aspect (T-avg), lateral aspect (T-avg) and medial aspect (T-avg). Overall, there were TI parameters which performed better than the rest, with some attaining an AUC results of > 0.70 to 0.80 or > 0.80.

**Table 2:** ROC analysis of TI parameters in identifying PD score>0 at the bilateral knees.

TI para- meter	Knee aspect	Optimal cut-off^#^	Youden’s index	SN (%)	SP (%)	PPV (%)	NPV (%)	AUC (95% CI)
Right knee (identifying PD score > 0)								
T-min	Lateral	29.6	0.329	63.2	69.7	34.3	88.3	0.67 (0.54, 0.79)
T-max	Lateral	33.5	0.526	73.7	78.9	46.7	92.3	0.76 (0.65, 0.88)
T-avg	Lateral	31.2	0.395	84.2	55.3	32.0	93.3	0.70 (0.60, 0.80)
T-min	Anterior	29.7	0.434	73.7	69.7	37.8	91.4	0.72 (0.60, 0.83)
T-max	Anterior	33.5	0.368	63.2	73.7	37.5	88.9	0.68 (0.56, 0.81)
T-avg	Anterior	31.2	0.447	84.2	60.5	34.8	93.9	0.72 (0.62, 0.82)
T-min	Medial	29.1	0.250	63.2	61.8	29.3	87.0	0.63 (0.50, 0.75)
T-max	Medial	33.5	0.447	63.2	81.6	46.2	89.9	0.72 (0.60, 0.84)
T-avg	Medial	30.3	0.303	94.7	35.5	26.9	96.4	0.65 (0.58, 0.73)
Left knee (identifying PD score > 0)								
T-min	Lateral	29.5	0.598	85.7	74.1	36.4	96.8	0.80 (0.69, 0.91)
T-max	Lateral	34.0	0.507	64.3	86.4	45.0	93.3	0.75 (0.62, 0.89)
T-avg	Lateral	31.6	0.598	85.7	74.1	36.4	96.8	0.80 (0.69, 0.91)
T-min	Anterior	30.7	0.460	57.1	88.9	47.1	92.3	0.73 (0.59, 0.87)
T-max	Anterior	33.6	0.539	78.6	75.3	35.5	95.3	0.77 (0.65, 0.89)
T-avg	Anterior	31.6	0.647	85.7	79.0	41.4	97.0	0.82 (0.72, 0.93)
T-min	Medial	29.3	0.511	85.7	65.4	30.0	96.4	0.76 (0.65, 0.86)
T-max	Medial	34.1	0.569	64.3	92.6	60.0	93.8	0.78 (0.65, 0.92)
T-avg	Medial	31.2	0.610	85.7	75.3	37.5	96.8	0.81 (0.70, 0.91)

*TI* Thermal imaging,* T-min* Minimum temperature, *T-max* Maximum temperature, *T-avg* Average temperature, *PD* Power Doppler, *SN* Sensitivity, *SP *Specificity, PPV Positive predictive value, *NPV *Negative predictive value, *AUC* Area under the receiving operating characteristic curve. ^#^Chosen based on Youden’s index.

**Table 3:** ROC analysis of TI parameters in identifying GS score ≥ 2 at the bilateral knees.

TI parameter	Knee aspect	Optimal cut-off^#^	Youden’s index	SN (%)	SP (%)	PPV (%)	NPV (%)	AUC (95% CI)
Right knee (identifying GS score ≥ 2)								
T-min	Lateral	29.6	0.383	66.7	71.6	40.0	88.3	0.69 (0.58, 0.81)
T-max	Lateral	33.5	0.512	71.4	79.7	50.0	90.8	0.76 (0.65, 0.87)
T-avg	Lateral	31.2	0.425	85.7	56.8	36.0	93.3	0.71 (0.62, 0.81)
T-min	Anterior	29.7	0.417	71.4	70.3	40.5	89.7	0.71 (0.60, 0.82)
T-max	Anterior	33.4	0.403	71.4	68.9	39.5	89.5	0.70 (0.59, 0.81)
T-avg	Anterior	31.2	0.479	85.7	62.2	39.1	93.9	0.74 (0.65, 0.83)
T-min	Medial	29.1	0.302	66.7	63.5	34.1	87.0	0.65 (0.53, 0.77)
T-max	Medial	33.5	0.382	57.1	81.1	46.2	87.0	0.69 (0.57, 0.81)
T-avg	Medial	30.3	0.317	95.2	36.5	29.9	96.4	0.66 (0.59, 0.73)
Left knee (identifying GS score ≥ 2)								
T-min	Lateral	29.7	0.566	73.7	82.9	51.9	92.6	0.78 (0.67, 0.89)
T-max	Lateral	33.7	0.434	68.4	75.0	40.6	90.5	0.72 (0.60, 0.84)
T-avg	Lateral	31.6	0.618	84.2	77.6	48.5	95.2	0.81 (0.71, 0.91)
T-min	Anterior	30.7	0.500	57.9	92.1	64.7	89.7	0.75 (0.63, 0.87)
T-max	Anterior	33.6	0.513	73.7	77.6	45.2	92.2	0.76 (0.64, 0.87)
T-avg	Anterior	31.5	0.632	84.2	78.9	50.0	95.2	0.82 (0.72, 0.91)
T-min	Medial	29.3	0.461	78.9	67.1	37.5	92.7	0.73 (0.62, 0.84)
T-max	Medial	34.1	0.461	52.6	93.4	66.7	88.8	0.73 (0.61, 0.85)
T-avg	Medial	31.2	0.632	84.2	78.9	50.0	95.2	0.82 (0.72, 0.91)

*TI* Thermal imaging, *T-min* Minimum temperature, *T-max* Maximum temperature,* T-avg* Average temperature, *GS* Grey-scale, *SN* Sensitivity, *SP *Specificity, *PPV* Positive predictive value, *NPV* Negative predictive value, *AUC* Area under the receiving operating characteristic curve. ^#^Chosen based on Youden’s index.

### Intra-observer reliability analysis

For intra-observer reliability analysis, Table 4 summarizes the ICC (and the 95% CI) results for the TI parameters at the knees of patients with RA. The ICC results were high for the TI parameters (T-min, T-max and T-avg) at the knees and ranged from 0.997 to 0.999.

**Table 4:** Intra-observer reliability for TI parameters at the knees.

TI parameter	Number of samples re-read (> 2 weeks apart)	ICC (95% CI)
T-min	30	0.997 (0.993, 0.998)
T-max	30	0.999 (0.9979, 0.9995)
T-avg	30	0.998 (0.997, 0.999)

*TI* Thermal imaging, *T-min* Minimum temperature, *T-max* Maximum temperature, *T-avg* Average temperature, *ICC *Intra-class correlation coefficient.

## Discussion

Previous RA studies have compared thermographic temperatures at the knees with ultrasound PD joint inflammation^10,11^. We have added to the RA imaging literature by comparing thermographic heat signatures at the knees of RA patients in relation to both ultrasound PD and GS joint inflammation. Through our study, we have demonstrated that thermographic temperatures are reflective of both ultrasound PD and GS joint inflammation and can help discriminate the absence/presence of PD vascularity and GS joint inflammation severity at the knees of patients with RA. In our correlation analysis, we have demonstrated that some TI parameters had a stronger correlation (with correlation coefficients between 0.40 and 0.50 indicating a moderate correlation) with ultrasound-detected joint inflammation. Similarly, for the ROC analysis at the knees, some of the TI parameters appear to perform better than the rest (with some attaining an AUC results of > 0.70 to 0.80 or > 0.80). Our study included TI from three aspects of the knee as the best aspect(s) of the knee to perform TI is presently unknown. In our study, not all TI parameters showing better correlation and higher AUC values are aggregated only at one aspect of the knee. Therefore, it appears that there is value to image all three aspects of the knee to allow for a more comprehensive assessment of the bilateral knees in patients with RA. The study by Vasdev et al.^10^ included 50 RA patients and 50 health controls and compared TI with PD ultrasound at the knees. The authors reported that the mean knee temperature and the mean knee–thigh temperature differential were both significantly greater in RA patients when compared to healthy controls. A separate study by Ahn et al.^11^ including 30 patients with knee arthritis (12 of whom had RA) demonstrated significantly higher knee temperature among those in the PD-positive group versus those in the PD-negative group, while the ROC analysis showed that the AUCs of thermographic temperatures in predicting PD positivity ranged between 0.764 and 0.790. Apart from ultrasound joint inflammation outcomes, RA studies have also shown that TI parameters at the knees were correlated with other clinical measures such as the C-reactive protein and erythrocyte sedimentation rate^23,24^. Taken together, the three studies (by Vasdev et al.^10^, Ahn et al.^11^ and our present study) suggest that TI is promising in the detection of ultrasound joint inflammation at the knees in patients with RA. Future studies should explore the use of thermography as an adjunctive tool to help in the screening of joint inflammation at the knees in patients with RA given that TI is less costly and requires less training for its operator when compared to musculoskeletal ultrasound^4,18,25^.

Unlike ultrasound which has been well validated by the European League Against Rheumatism-Outcome Measures in Rheumatology ultrasound workgroup for joint inflammation assessment in patients with RA^20,21^, there has been correspondingly less validation work (including reliability analysis) for TI in patients with RA. In one small-scale TI study (with various types of knee arthritis patients including RA)^11^, the inter-observer reliability for TI between two observers ranged from 0.973 to 0.999. In our present study with a single observer, the intra-observer reliability was found to be excellent as well (with ICC results > 0.90 for all the thermographic temperatures). Nonetheless, more studies ideally with multiple observers testing for intra- and inter-observer reliability of TI will be required at the knee (as well as other joint types) in patients with RA.

Our study is not without its limitations. Having a cross-sectional study design, thermography was compared with ultrasonography in patients with RA at a single time-point. Moreover, our study only includes joint inflammation outcomes without evaluating structural joint damage (such as bony erosions). Therefore, future prospective RA studies with a longitudinal study design with imaging performed at multiple time-points will be required to assess for the responsiveness of TI in monitoring joint inflammation over time; and ideally includes bone damage outcome measures (e.g. detected on conventional radiology, computed tomography and/or MRI) for comparative analysis. For thermography, our study included TI at the knee from three aspects (lateral, anterior and medial) as the best aspect(s) of the knee to perform TI is presently unknown. For ultrasonography, we chose ultrasound scanning of the knee at the suprapatellar recess since it is a representative site which also allows for a simple, fast and straightforward ultrasound imaging assessment. Future studies including more ultrasound locations at the knees would be necessary to help determine if (and how) this may affect the comparison between the two modalities. Another limitation of our current study is the absence of a control group (e.g. healthy subjects) to allow comparison of the differences in the TI parameters at the knees between the two groups. Future RA studies should ideally include a control group (e.g. healthy subjects) for comparison of the TI findings at the knees between the two groups.

## Conclusion

In summary, we have shown that thermographic heat signatures are reflective of both ultrasound PD and GS joint inflammation and can help discriminate the absence/presence of PD vascularity and GS joint inflammation severity at the knees of patients with RA. Our findings are consistent with data from the RA literature on ultrasound PD joint inflammation detection by TI at the knees. Additionally, our study shows that TI can be useful for ultrasound GS joint inflammation assessment. Furthermore, TI appears to be a reliable measure of joint inflammation at the RA knee. Overall, thermography appears promising, and our findings will need to be further validated in other independent RA cohorts.

## Data Availability

Data for this study are available from the corresponding author on reasonable request.
